# Identification and validation of an angiogenesis-related signature associated with preeclampsia by bioinformatic analysis

**DOI:** 10.1097/MD.0000000000032741

**Published:** 2023-02-03

**Authors:** Jiancai Ma, Hong Wu, Xiaofang Yang, Lulu Zheng, Haiqin Feng, Liping Yang

**Affiliations:** a Department of Obstetrics and Gynecology, Handan Central Hospital, Handan, China.

**Keywords:** CIBERSORT, GSEA, immune infiltration, preeclampsia (PE), risk signature

## Abstract

Preeclampsia (PE) is a pregnancy disorder with high morbidity and mortality rates for both mothers and newborns. This study explores potential diagnostic indicators of PE.

We downloaded the messenger ribonucleic acid profiles of the GSE75010 dataset from the Gene Expression Omnibus database, and used placenta samples to carry out different analyses including differential expression, Gene Ontology, and Kyoto Encyclopedia of Genes and Genomes analyses. Least absolute shrinkage and selection operator regression was constructed and the receiver operating characteristic curve was drawn to evaluate the accuracy of the model. An external validation was conducted to prove the stability of the risk model.

We found 140 angiogenesis-related genes and identified 29 angiogenesis-related genes between the 2 groups, including 12 upregulated genes and 17 downregulated genes. In addition, we established a 12-gene risk signature, which has a high accuracy in predicting PE during pregnancy (area under curve = 0.90). The immune infiltration characteristics are differentially distributed in the 2 groups, which may be the cause of hypertension during pregnancy. The external validation with the GSE25906 dataset confirmed the high accuracy of our model (area under curve = 0.87).

Our results outline the characteristics of a set of genes potentially involved in PE and its subgroups, contributing to a better understanding of the molecular mechanisms of PE.

## 1. Introduction

Preeclampsia (PE) is a pregnancy complication with distinct clinical subtypes. It affects up to 5% of pregnant women worldwide and is one of the leading causes of maternal and infant mortality.^[1,2]^ While the etiology of PE is still unclear, placental dysregulation is widely assumed to be the major cause of its pathogenesis.^[3]^ Timely identification of women at high risk of developing PE or severe PE and discovery of novel targets for drug therapy are crucial to avoid bad outcomes, yet there are currently no biomarkers to clinically predict the onset of PE. A recent study has demonstrated that dysregulated non-coding ribonucleic acids (RNAs) are involved in the pathogenesis of PE. A growing body of evidence suggests that the pathogenesis of PE may be related to angiogenesis (growth of new blood vessels from preexisting vasculature) and vascular remodeling (including branching, enlargement, and network formation).^[4]^

Endothelial cells in uterine arteries undergo proliferation, differentiation, and migration to establish a “low resistance, high receptivity” tissue, which increases uterine blood flow by 53 times during pregnancy.^[5,6]^ Both abnormal angiogenesis and reduced uterine blood flow can result in pregnancy disorders such as fetal growth restriction, stillbirth, gestational hypertension, preterm birth, miscarriage, and PE.^[7]^ The correct establishment and remodeling of blood vessels in uterine arteries is therefore crucial for fetal growth and survival. Understanding the mechanism behind angiogenesis would help researchers and clinicians formulating treatment strategies and alleviating clinical symptoms related to angiogenesis changes.^[8]^

Over the past 10 years, research has primarily focused on identifying transcriptome changes in PE placental tissue by microarrays and bioinformatics analyses, which help isolate key genes involved in PE pathogenesis.^[9]^ Gene expression profiling of PE has been included in the Gene Expression Omnibus (GEO) database. Gong et al has identified dysregulation genes from preeclamptic placenta tissues in a genome-wide expression analysis using high-density oligonucleotide microarrays.^[10]^

In this study, we investigated the function of angiogenesis-related genes (ARGs) and constructed a risk signature to predict PE by integrating bioinformatic analysis and immune infiltration comparison. Gene Ontology (GO), Kyoto Encyclopedia of Genes and Genomes (KEGG) pathway enrichment analysis, and Gene Set Enrichment Analysis (GSEA) were subsequently performed to help us understand the molecular mechanisms underlying PE pathogenesis. The accuracy and stability of the selected genes and of our risk model in predicting the risk of PE during pregnancy are both high. Our results can therefore contribute to a better understanding of PE pathogenesis and provide a rationale for the development of effective diagnostic or therapeutic strategies.

## 2. Materials and methods

### 2.1. Data download and identification of ARGs

We obtained publicly available gene expression data of PE samples from GEO (http://www.ncbi.nlm.nih.gov/gds/) as series matrix files. We downloaded the gene expression profiling dataset GSE75010, based on GPL6244. The dataset consists of placenta expression profiles of 2 groups (80 PE placentas and 77 non-PE placentas). Expression data were then utilized to identify differentially expressed genes (DEGs) in the dataset after normalization using the “*limma*” R package. The angiogenesis-related DEGs set was downloaded from the Molecular Signature Database using the GSEA tool (http://www.gsea-msigdb.org/gsea/msigdb/human/search.jsp). ARGs between PE patients and controls were determined setting *P* < .05 and |log 2 FC| > 1 as having significantly differential expressions. The approval of Ethics Committee isn’t necessary in our study.

### 2.2. Functional enrichment analysis

GO and KEGG pathway enrichment analysis were performed with the “*cluster-Profiler*” R package to investigate potential functions of DEGs and analyze enrichment regarding biological processes, cellular components, and molecular functions.^[11,12]^ *P* < .05 was set as threshold value after being adjusted with the Benjamini–Hochberg method.

### 2.3. Derivation of ARGs signature and risk classifier

In this study, we used the least absolute shrinkage and selection operator (LASSO) regression model^[13]^ to select the most predictive genes from the identified ARGs, with 10-fold cross-validation and 1000 iterations to stabilize the results in which λ was determined by the 1-SE criterion. We then calculated the risk score of each gene as the sum of the gene expression level and its corresponding coefficient derived from LASSO: risk score=∑(coefficient i × expression of signature gene i). Based on the risk score, we determined the optimal cutoff value with the maximum selection rank statistical method in order to develop a risk classifier that distinguishes PE patients and controls. The reliability of the selected genes and of the predictive model was validated by receiver operating characteristic (ROC) analysis.

### 2.4. GSEA and CIBERSORT analyses

Functional differences between patients with high and low risk scores were identified based on GSEA annotation from the “*clusterProfiler*” package. Differences among risk groups were verified by stromal score, immune score, and ESTIMATE score calculated by the “*estimate*” package in R (https://cran.r-project.org/src/contrib/Archive/).^[14]^ Scores between 2 groups were compared with the Mann–Whitney *U* test. Relative proportion and distribution of 22 immune cell types in each sample of a subset of the GSE75010 dataset were assessed using the CIBERSORT software. CIBERSORT is a tool that characterizes the immune cell composition of 547 preset barcoded genes based on gene expression profiles and a deconvolution algorithm, then sums the relative percentages of estimated immune cell types in each sample to 1. The box plot was constructed based on the proportion of immune cells in different risk groups.^[15]^ Expression level of human leukocyte antigens and checkpoint-related genes in 2 subgroups were compared by Mann–Whitney test.

### 2.5. Correlation analysis between hub genes

We used the “*corplot*” R packages to analyze the correlations between selected genes, and calculated the Pearson correlation coefficients and *P* values.

### 2.6. External validation of the risk model

In order to verify the messenger RNA expression levels of the selected genes, we obtained expression data of the PE and control group from the GEO database (accession number: GSE25906). The expression data consisted of 23 PE patients and 37 control individuals. We downloaded the data matrix of the PE group and of the control group. We validated the expression of the selected genes in different groups and calculated the risk score of each patient, we then confirmed the reliability of the predictive model with a ROC curve.

### 2.7. Statistical analysis

Statistical data were analyzed using the R software (v3.5.2, http://www.R-project.org, The R Foundation, accessed on 1 June 2021). Continuous results were examined by *t* tests and categorical results by chi-squared tests. Spearman correlation and bi-directional were calculated using the function cor. test, *P* < .05 was considered statistically significant.

## 3. Results

### 3.1. Functional annotation of ARGs

**Figure 1.**
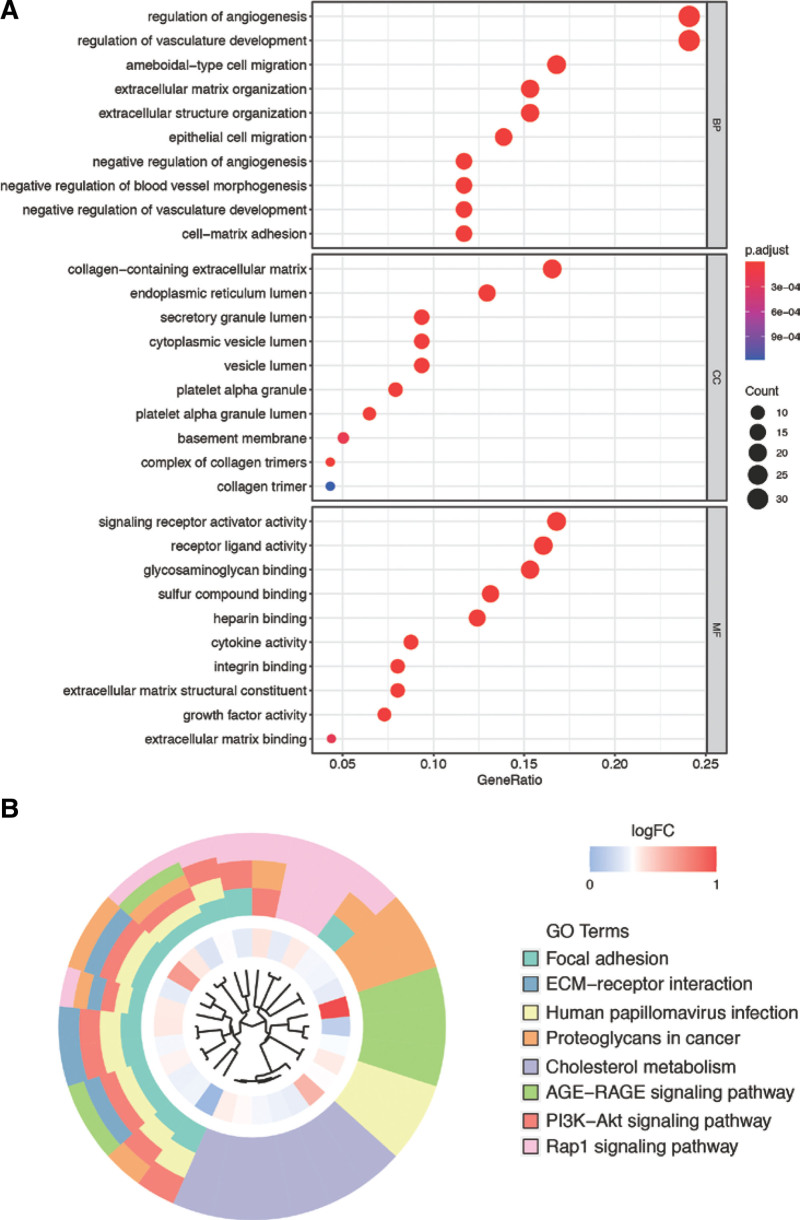
Functional enrichment results of angiogenesis-related genes. (A) GO bubble plot. (B) KEGG analysis. GO = Gene Ontology, KEGG = Kyoto Encyclopedia of Genes and Genomes.

In order to get insights into the functions of ARGs, we carried out GO and KEGG enrichment analyses. GO analysis showed ARGs were mainly enriched in regulation of angiogenesis, collagen-containing extracellular matrix (ECM), and signaling receptor activator activity (Fig. 1A). KEGG results showed these genes were enriched in focal adhesion, ECM–receptor interaction, and human papillomavirus infection pathways (Fig. 1B). These results suggested that the genes are enriched in angiogenesis-related functions, and these functions may play an important role in the occurrence of PE.

### 3.2. Differential expression of ARGs between PE and control groups

**Figure 2.**
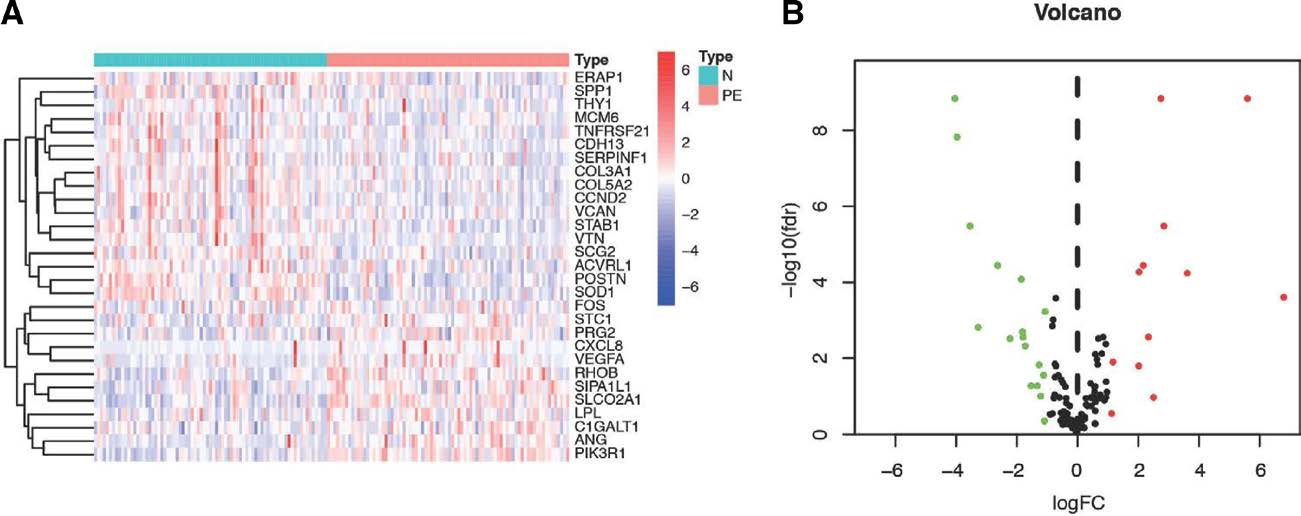
Plots of differentially expressed genes. (A) Volcano plot of the differentially expressed angiogenesis-related genes. (B) The differentially expressed upregulated and downregulated angiogenesis-related genes were selected to compare their expression differences by heatmap.

ARGs between placentas of PE patients and controls were analyzed using the *limma* R package. ARGs were determined as follows: adjusted *P* < .05 and |log2 − fold change| >1. 29 ARGs were identified between the 2 groups, 12 upregulated genes and 17 downregulated genes, as shown in the heatmap and volcano plot in Figure 2A and B. These DEGs may play a pivotal role in the progress of PE.

### 3.3. Construction of the angiogenesis-based gene signature

**Figure 3.**
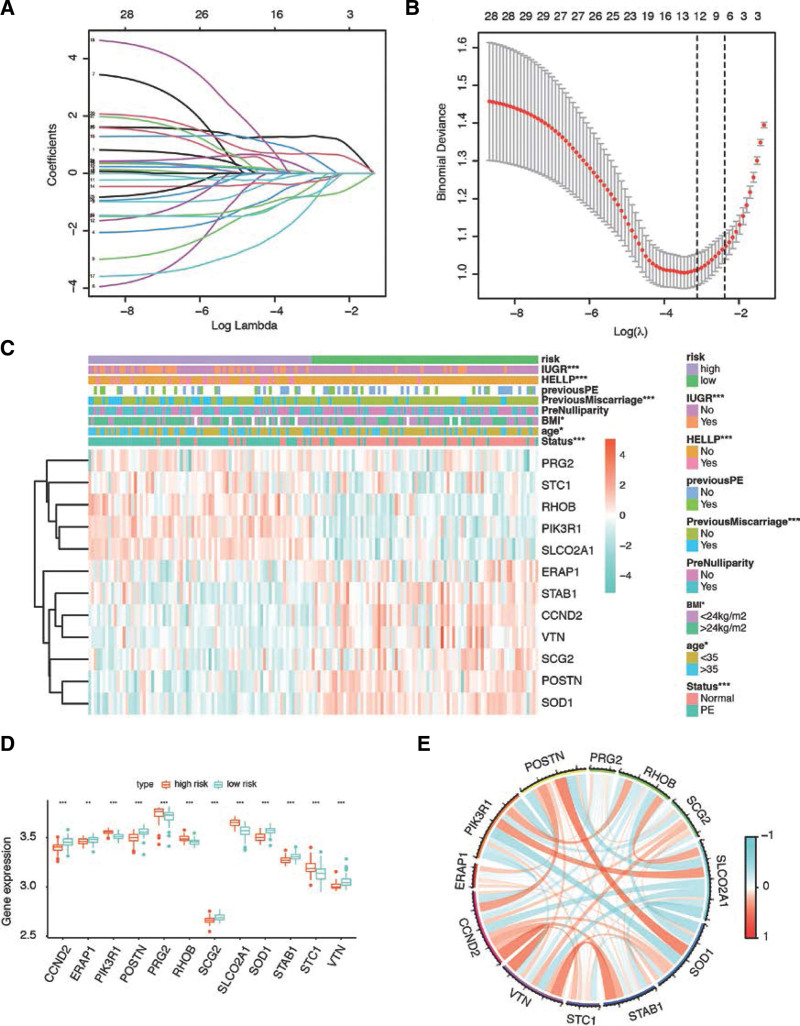
Validation of the angiogenesis-based classifier in GSE75010 subgroups for predicting PE. (A) LASSO coefficient profiles of the angiogenesis-based DEGs. The dotted line indicates the value chosen by 10-fold cross-validation. (B) Ten-fold cross-validation for tuning parameter selection in the LASSO model. The partial likelihood deviance is plotted against log (λ), where λ is the tuning parameter. Partial likelihood deviance values are shown, with error bars representing SE. (C) Heatmap showed the expression of the 12 angiogenesis-related hub genes in high- and low-risk patients in the GSE75010 dataset associated with IUGR, HELLP, previous PE, previous miscarriage, pre-nulliparity, BMI, age, and PE status. *P < .05; **P < .01; ***P < .001. (D) Expression levels of 12 significant hub genes in high- and low-risk groups. (E) Correlation analysis between 12 hub genes. BMI = body mass index, DEGs = differentially expressed genes, HELLP = hemolysis, elevated liver function and low platelet count, IUGR = intrauterine growth retardation, LASSO = least absolute shrinkage and selection operator, PE = preeclampsia, SE = standard error.

In order to avoid over-fitting the prognostic signature, the LASSO regression model was employed to select the most useful gene signature from all ARGs within the cohort (Fig. 3A and B). We identified the optimal gene signature consisting of 12 ARGs (PRG2, STC1, RHOB, PIK3R1, SLCO2A1, ERAP1, STAB1, CCND2, VTN, SCG2, POSTN, and SOD1) and their corresponding coefficients (Table 1). The risk score of each patient was calculated based on the expression levels of 12 ARGs and their coefficients as: risk score = −0.967 × CCND2 −1.767 × ERAP1 + 1.262 × PIK3R1 −0.623 × POSTN + 0.202 × PRG2 + 1.637 × RHOB −3.273 × SCG2 + 0.636 × SLCO2A1 −0.905 × SOD1 −1.200 × STAB1 + 1.056 × STC1 −0.370 × VTN.

**Table 1:** Twelve angiogenesis-associated genes and corresponding coefficient value.

Angiogenesis-related gene name	Coefficient
CCND2	−0.967
ERAP1	−1.767
PIK3R1	1.262
POSTN	−0.623
PRG2	0.202
RHOB	1.637
SCG2	−3.273
SLCO2A1	0.636
SOD1	−0.905
STAB1	−1.200
STC1	1.056
VTN	−0.370
Risk score	Low: <6.62
	High: ≥6.62

We developed a predictive classifier based on the median risk score to classify patients into high- and low-risk groups. In addition, we generated a heatmap that shows the expression characters of the 12 selected genes (Fig. 3C). We observed significant differences between the 2 groups associated with intrauterine growth retardation, hemolysis, elevated liver function and low platelet count, previous PE, previous miscarriage, BMI, age, and PE status. We visualized the expressions of the 12 genes in the high- and low-risk groups as a box plot (Fig. 3D). The results of correlation analysis were shown in Figure 3E. We tested the enrichment in biological pathways by GSEA. We found that genes in patients with high risk scores were enriched in B cell receptor signaling pathway, chemokine signaling pathway, citrate tricarboxylic acid cycle, cytokine–cytokine receptor interaction, and focal adhesion. The genes of low risk patients were enriched in mismatch repair, natural killer (NK) cell-mediated cytotoxicity, pyruvate metabolism, steroid biosynthesis, and transforming growth factor-β signaling pathway (Figure S1, Supplemental Digital Content, http://links.lww.com/MD/I350). We identified 24 dysregulated microRNA (miRNA) regulators of hub genes. Related miRNAs for targeted genes and its connections are shown in Figure S2, Supplemental Digital Content, http://links.lww.com/MD/I351. These results indicated that the ARGs and risk model are predictive for PE.

### 3.4. Evaluation of predictive accuracy of the 12 selected genes by ROC curve

**Figure 4.**
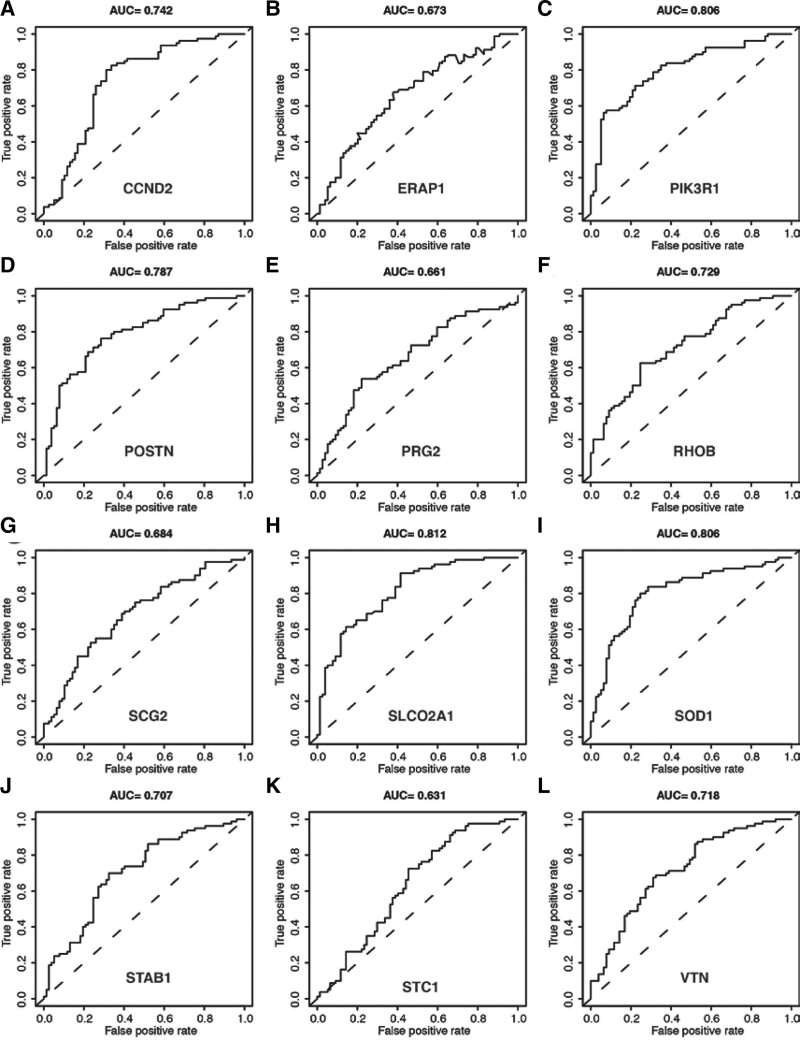
Predictive accuracy of 12 angiogenesis-related hub gene for PE. (A) CCND2. (B) ERAP1. (C) PIK3R1. (D) POSTN. (E) PRG2. (F) RHOB. (G) SCG2. (H) SLCO2A1. (I) SOD1. (J) STAB1. (K) STC1. (L) VTN. PE = preeclampsia.

In order to confirm the accuracy of the 12 selected genes, we plotted the ROC curves of the model. The area under curve (AUC) values were 0.742 (CCND), 0.673 (ERAP1), 0.806 (PIK3R1), 0.787 (POSTN), 0.661 (PRG2), 0.729 (RHOB), 0.684 (SCG2), 0.812 (SLCO2A1), 0.806 (SOD1), 0.707 (STAB1), 0.631 (STC1), and 0.718 (VTN), as shown in Figure 4. These results suggest that the genes are highly accurate in predicting PE in pregnant women (AUC > 0.6), especially PIK3R1, SLCO2A1, and SOD1 (AUC > 0.8).

### 3.5. Validation of the ARG-based risk model and immune characteristics in 2 classifiers

**Figure 5.**
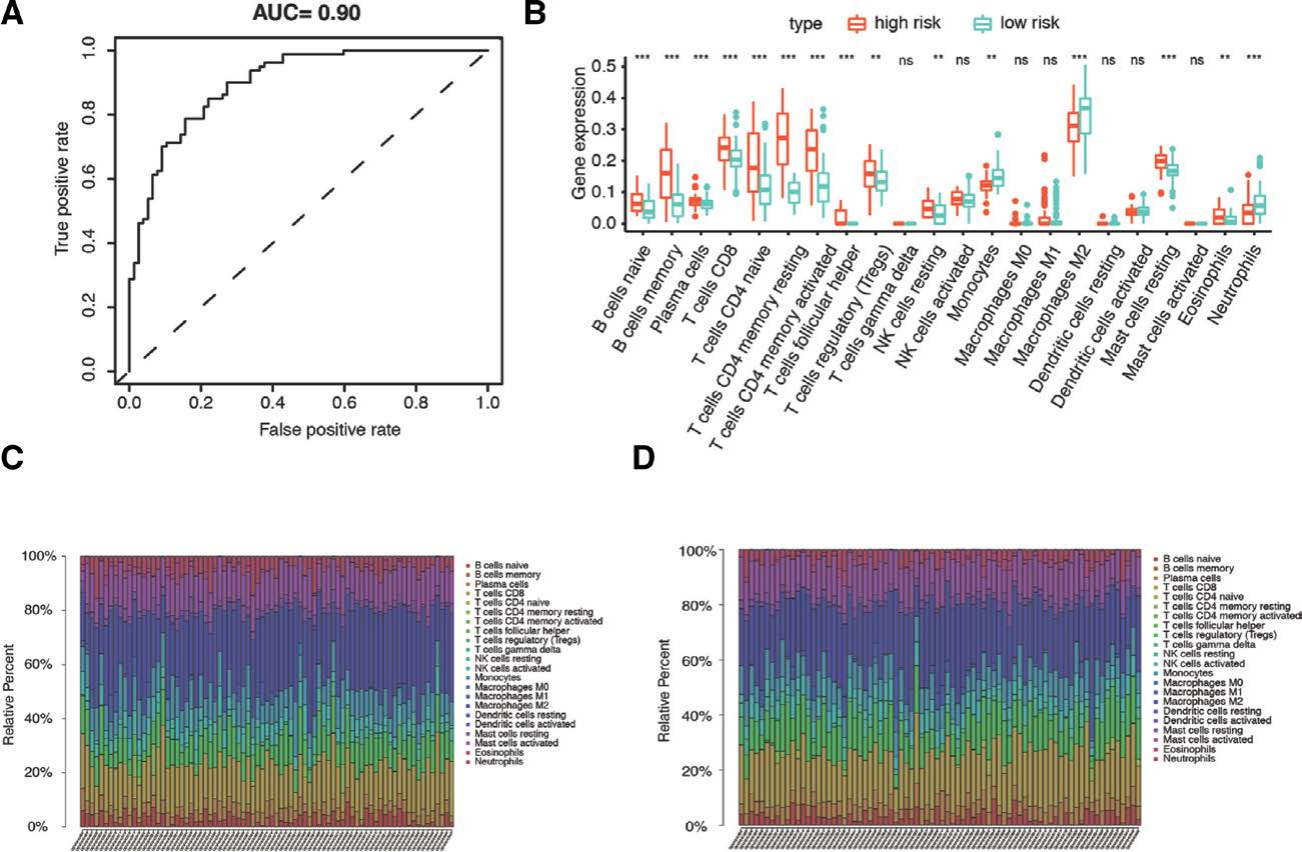
Immune cell infiltration in different subgroups. (A) ROC curve of the risk signature for predicting PE. (B) The difference of immune cell infiltration between low and high-risk groups (Wilcoxon test). (C) The immune cell infiltration in low-risk samples and (D) high-risk samples. PE = preeclampsia, ROC = receiver operating characteristic.

In order to validate the accuracy of risk scores in predicting PE, we constructed the ROC curve. The AUC of the risk model for predicting PE was 0.90 (Fig. 5A). In addition, we explored the immune microenvironment using the CIBERSORT algorithm to estimate the relative abundance of 22 types of immune cells for patients in the high- and low-risk groups. In the box plot in Figure 5B the relative percentages of 22 immunocyte subtypes in each patient are visualized in different colors. Results show the immune infiltration in low-risk samples is significantly different from high-risk samples (*P* < .05). Based on the Wilcoxon rank-sum test, resting B cells, T cells, CD4 memory activated cells, T follicular helper cells, T cells regulatory cells, and resting NK cells, had higher presence in the high-risk group. In contrast, monocytes, M2 macrophages, resting mast cells, eosinophils, and neutrophils accounted for a lower fraction in the high-risk group, indicating immunocyte infiltration was promoted in the high-risk group. As shown in Figure 5C and D, the immune characteristics analysis had similar results, with more immune infiltrations found in samples with high risk scores or low placenta purity. The low-risk group displayed significantly lower expressions of human leukocyte antigens-related genes (Figure S3, Supplemental Digital Content, http://links.lww.com/MD/I352). These results suggested that the model has a good effect in differentiating the 2 subgroups in terms of immunity.

### 3.6. External validation of the risk score model

**Figure 6.**
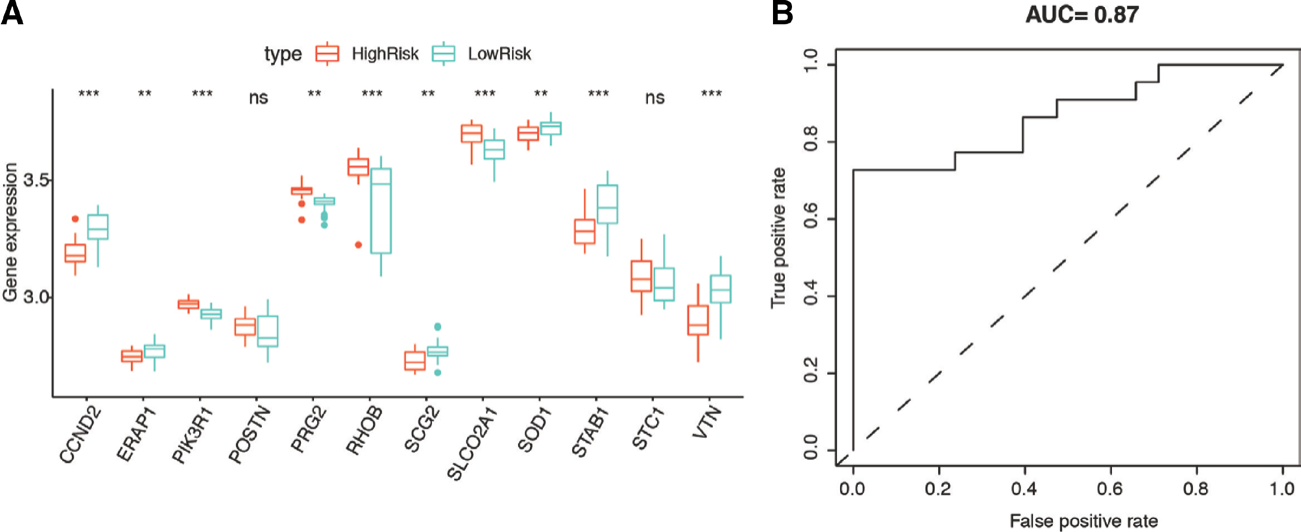
Validation of the risk signature in GSE25906. (A) Expression of 12 hub genes in different groups. (B) ROC curve of the risk model. ROC = receiver operating characteristic.

We conducted the external validation with dataset GSE25906. The expression of the 12 genes we considered corresponded with that in GSE75010, except for POSTN and STC1 (Fig. 6A).

We plotted the ROC curves to confirm the model accuracy (Fig. 6B). The AUC value in the validation set (GSE25906) was 0.87, indicating that the ARGs-based risk model effectively separates pregnant patients with hypertension from healthy individuals.

## 4. Discussion

PE has high morbidity and is a leading cause of maternal and fetal mortality across the world.^[16]^ Fetal malformation, preterm birth, and unexpected complications caused by PE have high economic and psychological costs both for the patient’s family and society. We know the placenta is an important site for PE pathogenesis. Identifying genes that have different expression levels between normal and preeclamptic placentas can help us identify effective biomarkers and therapeutic targets, and can also improve our understanding of the molecular mechanisms involved in PE pathogenesis.

Here, we used the R package *clusterProfiler* to carry out enrichment analysis of 140 angiogenesis-related gene. Results found several vascular related functions or pathways, such as regulation of angiogenesis, regulation of vasculature development, epithelial cell migration, and ECM–receptor interaction, were enriched in this gene set. Consistently with previous studies,^[17]^ our results also demonstrated that angiogenesis-related proteins can be used as early diagnostic markers of PE. It has been reported in literature that δ-TT may be hazardous to pregnancy because it can affect the regulation of epithelial cell migration and should therefore not be used in preeclamptic patients.^[18]^ Recent studies have suggested that impaired trophoblast function can lead to PE, and human trophoblast cell migration/invasion is tightly regulated by the ECM.^[19]^

Despite increasing efforts to mine databases to identify molecular markers that can help diagnose and treat PE, ARGs in PE and immune infiltrates had not yet been investigated. Substantial evidence indicates that vascular disorder and deficient trophoblast invasion are involved in PE pathogenesis.^[20,21]^ Angiogenesis is key in pregnancy for remodeling and enhancing vasodilation of maternal uterine arteries, and for increasing uterine blood flow. Both abnormal angiogenesis and decreased uteroplacental blood flow have been reported to be associated with the development of pregnancy disorders.^[22]^

In this study, we analyzed the expression profiles of 140 ARGs in placenta samples of patients with PE and healthy individuals by filtering gene chip data downloaded from GEO. We screened a total of 29 DEGs, which may be associated with the occurrence of PE during pregnancy. GO and KEGG pathway enrichment analysis showed significantly enriched GO terms and pathways which helped us to get a better understanding of PE pathogenesis. In addition, we performed LASSO regression analysis and identified key genes to construct a risk signature to predict PE in pregnant women. We investigated the accuracy of the selected genes and risk model and compared immune infiltration characteristics between the 2 groups.

PE patients were divided into 5 clusters based on unsupervised clustering of hub genes, in which the first and second clusters showed significantly different immune cell infiltration and gene expression. This demonstrated the hub gene can be used to classify PE into different subtypes.^[23]^ In addition, another study has investigated microarray data mining processes based on bioinformatics methods and constructed long non-coding RNA and miRNA networks for 10 hub genes closely related to PE.^[24]^

Compared with other risk models, the signature we constructed shows high accuracy in distinguishing healthy individuals and PE patients. The LASSO regression identified 12 hub genes, not yet investigated in detail in existing literature. Some of these genes have been demonstrated to exert essential roles in the pathogenesis of PE. PRG2, which encodes the major basic protein of eosinophil, is one of the most highly expressed genes during human pregnancy, and low PRG2 levels can predict both Down syndrome and PE.^[25]^ As both low oxygen and cAMP are known to play a central role in placental function, their regulation of STC-1 points to a potential role of this gene in the protection from prolonged placental hypoxia seen in PE. In our study we observed the expression of genes including VTN, CCND2, RHOB, PIK3R1, and SOD1 were consistent with what had been reported in other studies.^[26,27]^ These findings may provide potential biomarkers for hypertension in pregnancy.

Further analysis found patients in high risk groups were enriched in B cell receptor signaling pathway, chemokine signaling pathway, focal adhesion, and mismatch repair. One study has revealed that hypoxia-induced HIF-1α regulated Notch1/ETBR signaling, thereby modulating invasion and angiogenesis of trophoblast cells through immune cells.^[28]^ Cytokines/chemokines are involved in inflammatory events that induce trophoblast invasion during the development of PE.^[29]^

Immune cell infiltration analysis, a new bioinformatics strategy, has been successfully applied in the diagnosis and prognosis of several diseases.^[30]^ We analyzed immune cell infiltration in the high- and low-risk groups with CIBERSORT. We found significantly different infiltration between high- and low-risk groups for 15 types of immune cell: naïve B cells, plasma cells, naïve T cells CD4, CD4 memory resting T cells, follicular helper T cells, regulatory T cells, resting NK cells, CD4 memory activated T cells, monocytes, M2 macrophages, B memory cells, CD8 T cells, resting mast cells, eosinophils, and neutrophils. This further demonstrates immune cell infiltration is crucial in the pathogenesis and development of PE.

We acknowledge our study has some limitations. First, although 12 essential genes were identified as potential biomarkers for PE, further experimental verification should be performed on placenta tissues by quantitative polymerase chain reaction analysis. Second, the results of this study need to be further confirmed at molecular level, so that mechanisms underlying PE pathogenesis in the high risk group can be fully characterized.

## 5. Conclusion

In conclusion, based on publicly accessible data from the GEO database, we identified ARGs by bioinformatic analysis; these genes may play key roles in hypertension in pregnancy. In addition, we constructed a prognostic signature based on these genes. Our results indicate that 2 risk groups (high and low risk) have significant differences in immune cell infiltration and hub gene expression, suggesting ARGs can be used to predict PE with substantial accuracy.

## Author contributions

**Conceptualization:** Jiancai Ma.

**Data curation:** Hong Wu.

**Formal analysis:** Jiancai Ma.

**Investigation:** Hong Wu.

**Methodology:** Liping Yang.

**Project administration:** Xiaofang Yang.

**Resources:** Xiaofang Yang.

**Software:** Lulu Zheng.

**Supervision:** Lulu Zheng.

**Validation:** Haiqin Feng.

**Writing-original draft:** Jiancai Ma.

**Writing-review & editing:** Jiancai Ma, Liping Yang.

## Supplementary Material









## References

[1] Burton GJ Redman CW Roberts JM. Pre-eclampsia: pathophysiology and clinical implications. BMJ. 2019;366:l2381. 31307997 10.1136/bmj.l2381

[2] Phipps EA Thadhani R Benzing T. Pre-eclampsia: pathogenesis, novel diagnostics and therapies. Nat Rev Nephrol. 2019;15:275–89. 30792480 10.1038/s41581-019-0119-6 PMC6472952

[3] Jia N Li J. Role of circular RNAs in preeclampsia. Dis Markers. 2019;2019:17237495–7. 10.1155/2019/7237495 PMC6525895 31191755

[4] Schrey-Petersen S Stepan H. Anti-angiogenesis and preeclampsia in 2016. Curr Hypertens Rep. 2017;19:6. 28155021 10.1007/s11906-017-0706-5

[5] Motta AB. The role of obesity in the development of polycystic ovary syndrome. Curr Pharm Des. 2012;18:2482–91. 22376149 10.2174/13816128112092482

[6] Aneman I Pienaar D Suvakov S. Mechanisms of key innate immune cells in early- and late-onset preeclampsia. Front Immunol. 2020;11:1864. 33013837 10.3389/fimmu.2020.01864 PMC7462000

[7] Ananth CV. Ischemic placental disease: a unifying concept for preeclampsia, intrauterine growth restriction, and placental abruption. Semin Perinatol. 2014;38:131–2. 24836823 10.1053/j.semperi.2014.03.001

[8] Rana S Lemoine E Granger JP. Preeclampsia: pathophysiology, challenges, and perspectives. Circ Res. 2019;124:1094–112. 30920918 10.1161/CIRCRESAHA.118.313276

[9] Liang M Niu J Zhang L. Gene expression profiling reveals different molecular patterns in G-protein coupled receptor signaling pathways between early- and late-onset preeclampsia. Placenta. 2016;40:52–9. 27016783 10.1016/j.placenta.2016.02.015

[10] Gong S Gaccioli F Dopierala J. The RNA landscape of the human placenta in health and disease. Nat Commun. 2021;12:2639. 33976128 10.1038/s41467-021-22695-y PMC8113443

[11] Yu G Wang LG Han Y. clusterProfiler: an R package for comparing biological themes among gene clusters. OMICS. 2012;16:284–7. 22455463 10.1089/omi.2011.0118 PMC3339379

[12] Walter W Sanchez-Cabo F Ricote M. GOplot: an R package for visually combining expression data with functional analysis. Bioinformatics. 2015;31:2912–4. 25964631 10.1093/bioinformatics/btv300

[13] Tibshirani R. The LASSO method for variable selection in the Cox model. Stat Med. 1997;16:385–95. 9044528 10.1002/(sici)1097-0258(19970228)16:4<385::aid-sim380>3.0.co;2-3

[14] Yoshihara K Shahmoradgoli M Martinez E. Inferring tumour purity and stromal and immune cell admixture from expression data. Nat Commun. 2013;4:2612. 24113773 10.1038/ncomms3612 PMC3826632

[15] Newman AM Liu CL Green MR. Robust enumeration of cell subsets from tissue expression profiles. Nat Methods. 2015;12:453–7. 25822800 10.1038/nmeth.3337 PMC4739640

[16] Mol BWJ Roberts CT Thangaratinam S. Pre-eclampsia. Lancet. 2016;387:999–1011. 26342729 10.1016/S0140-6736(15)00070-7

[17] Shangguan Y Wang Y Shi W. Systematic proteomics analysis of lysine acetylation reveals critical features of placental proteins in pregnant women with preeclampsia. J Cell Mol Med. 2021;25:10614–26. 34697885 10.1111/jcmm.16997 PMC8581308

[18] Shi M Chen X Li H. Delta-tocotrienol suppresses the migration and angiogenesis of trophoblasts in preeclampsia and promotes their apoptosis via miR-429/ ZEB1 axis. Bioengineered. 2021;12:1861–73. 34002673 10.1080/21655979.2021.1923238 PMC8806315

[19] Liu C Hu Y Wang Z. The downregulation of placental lumican promotes the progression of preeclampsia. Reprod Sci. 2021;28:3147–54. 34231169 10.1007/s43032-021-00660-w PMC8526455

[20] Raymond D Peterson E. A critical review of early-onset and late-onset preeclampsia. Obstet Gynecol Surv. 2011;66:497–506. 22018452 10.1097/OGX.0b013e3182331028

[21] Boulanger H Flamant M. [New insights in the pathophysiology of preeclampsia and potential therapeutic implications]. Nephrol Ther. 2007;3:437–48. 18047998 10.1016/j.nephro.2007.07.001

[22] Mishra JS Chen DB Kumar S. AT2R activation increases in vitro angiogenesis in pregnant human uterine artery endothelial cells. PLoS One. 2022;17:e0267826. 35486619 10.1371/journal.pone.0267826 PMC9053770

[23] Meng Y Li C Liu CX. Immune cell infiltration landscape and immune marker molecular typing in preeclampsia. Bioengineered. 2021;12:540–54. 33535891 10.1080/21655979.2021.1875707 PMC8806319

[24] Wang C Yang C Wang X. ceRNA network and functional enrichment analysis of preeclampsia by weighted gene coexpression network analysis. Comput Math Methods Med. 2022;2022:15052354–14. 10.1155/2022/5052354 PMC8759911 35035521

[25] Weyer K Glerup S. Placental regulation of peptide hormone and growth factor activity by proMBP. Biol Reprod. 2011;84:1077–86. 21270431 10.1095/biolreprod.110.090209

[26] Wang Z Cai B Cao C. Downregulation of CD151 induces oxidative stress and apoptosis in trophoblast cells via inhibiting ERK/Nrf2 signaling pathway in preeclampsia. Free Radic Biol Med. 2021;164:249–57. 33450381 10.1016/j.freeradbiomed.2020.12.441

[27] Song J Li Y An RF. Identification of early-onset preeclampsia-related genes and MicroRNAs by bioinformatics approaches. Reprod Sci. 2015;22:954–63. 25717061 10.1177/1933719115570898

[28] Yu N Wu JL Xiao J. HIF-1alpha regulates angiogenesis via Notch1/STAT3/ETBR pathway in trophoblastic cells. Cell Cycle. 2019;18:3502–12. 31724455 10.1080/15384101.2019.1689481 PMC6927703

[29] Zhang X Wei H. Role of decidual natural killer cells in human pregnancy and related pregnancy complications. Front Immunol. 2021;12:728291. 34512661 10.3389/fimmu.2021.728291 PMC8426434

[30] Wang Y Li Z Song G. Potential of immune-related genes as biomarkers for diagnosis and subtype classification of preeclampsia. Front Genet. 2020;11:579709. 33335538 10.3389/fgene.2020.579709 PMC7737719

