# Supplementary figures and images for: Identification and validation of an angiogenesis-related signature associated with preeclampsia by bioinformatic analysis
Source: Medicine (Baltimore). 2023 Feb 3;102(5):e32741. doi: 10.1097/MD.0000000000032741 (PMC9902003; doi:10.1097/MD.0000000000032741)

## Slide 1
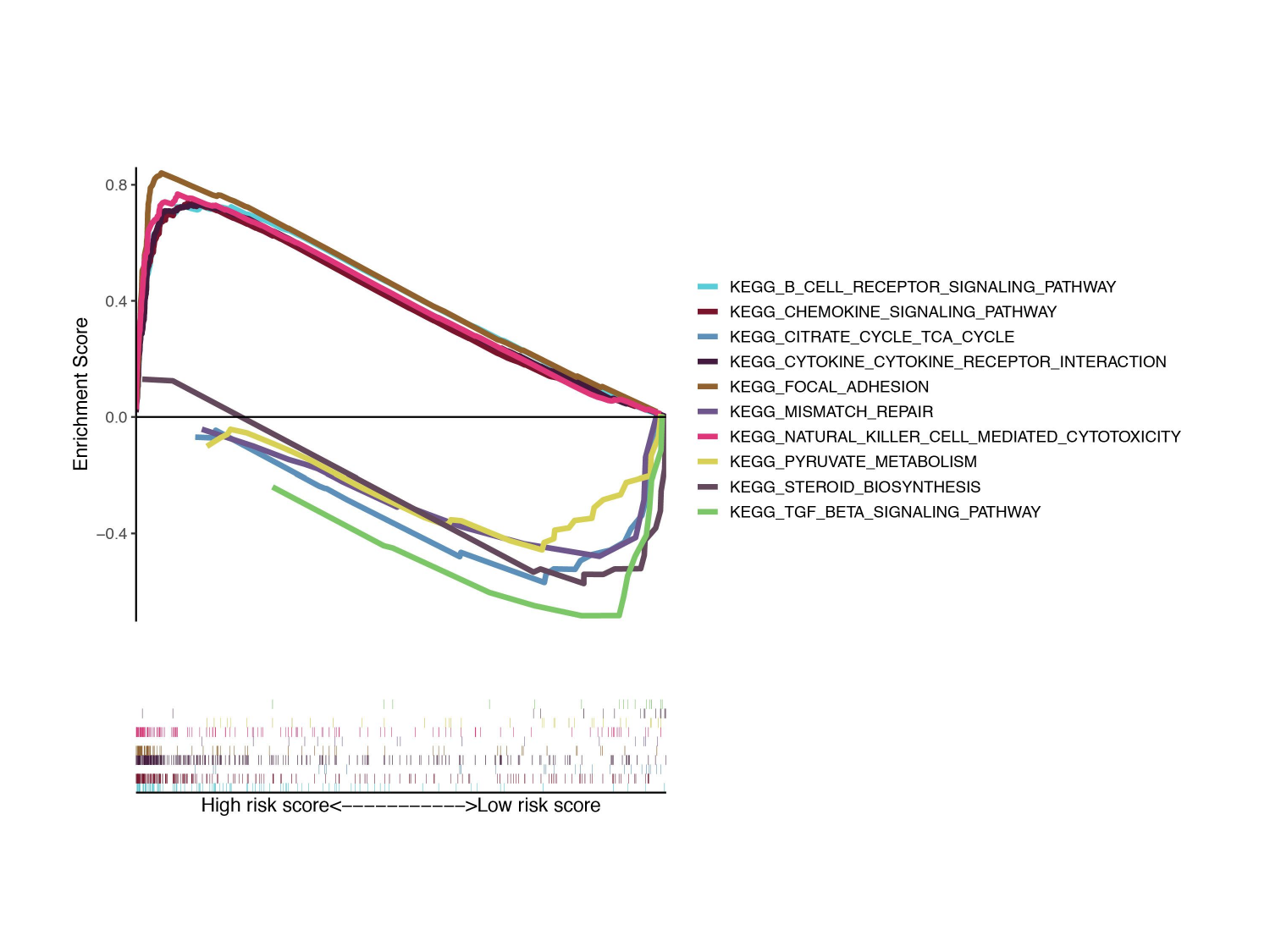

Supplement: Supplementary file 1 [file medi-102-e32741-s001.pptx]

## Slide 1
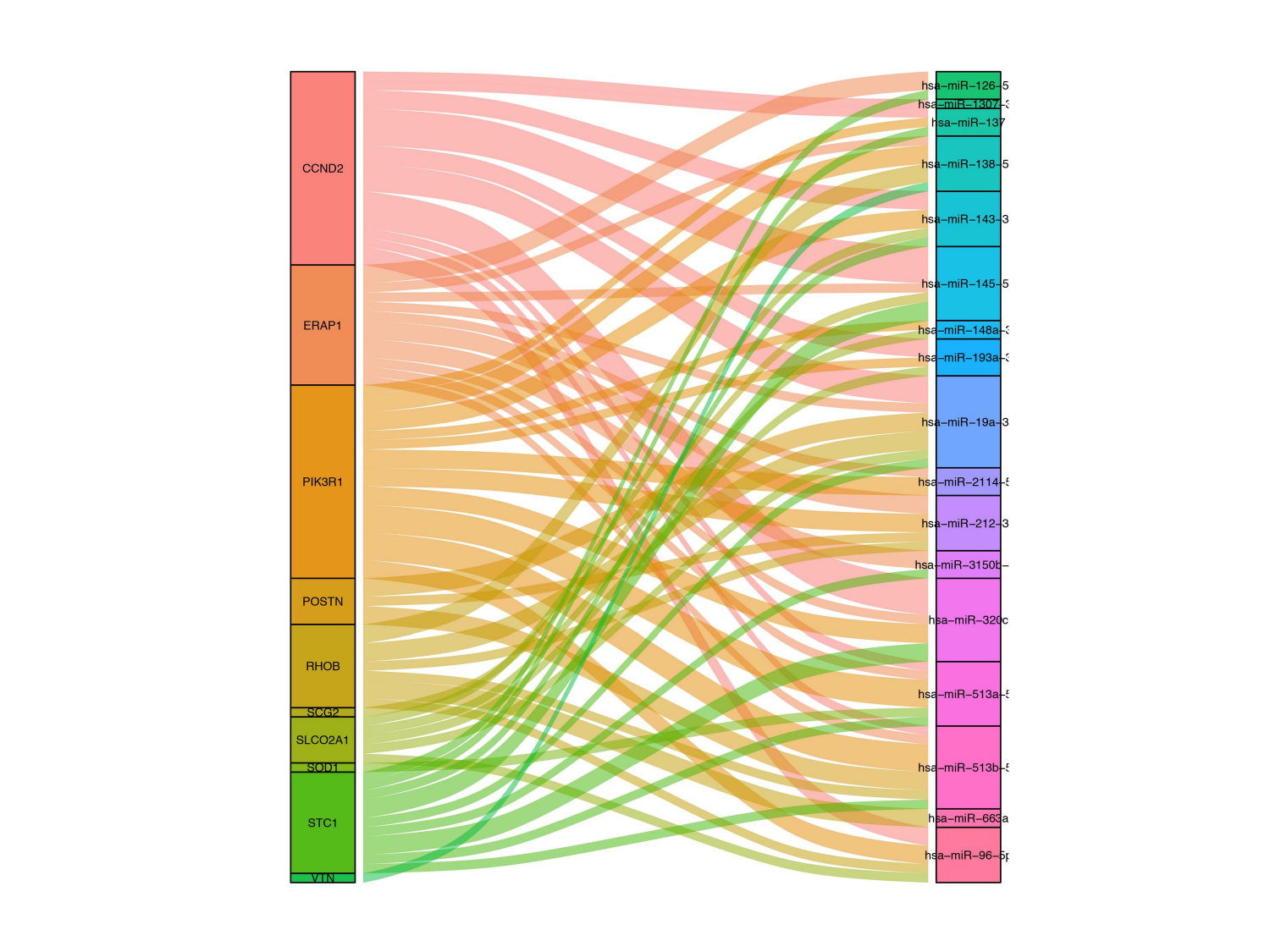

Supplement: Supplementary file 2 [file medi-102-e32741-s002.pptx]

## Slide 1
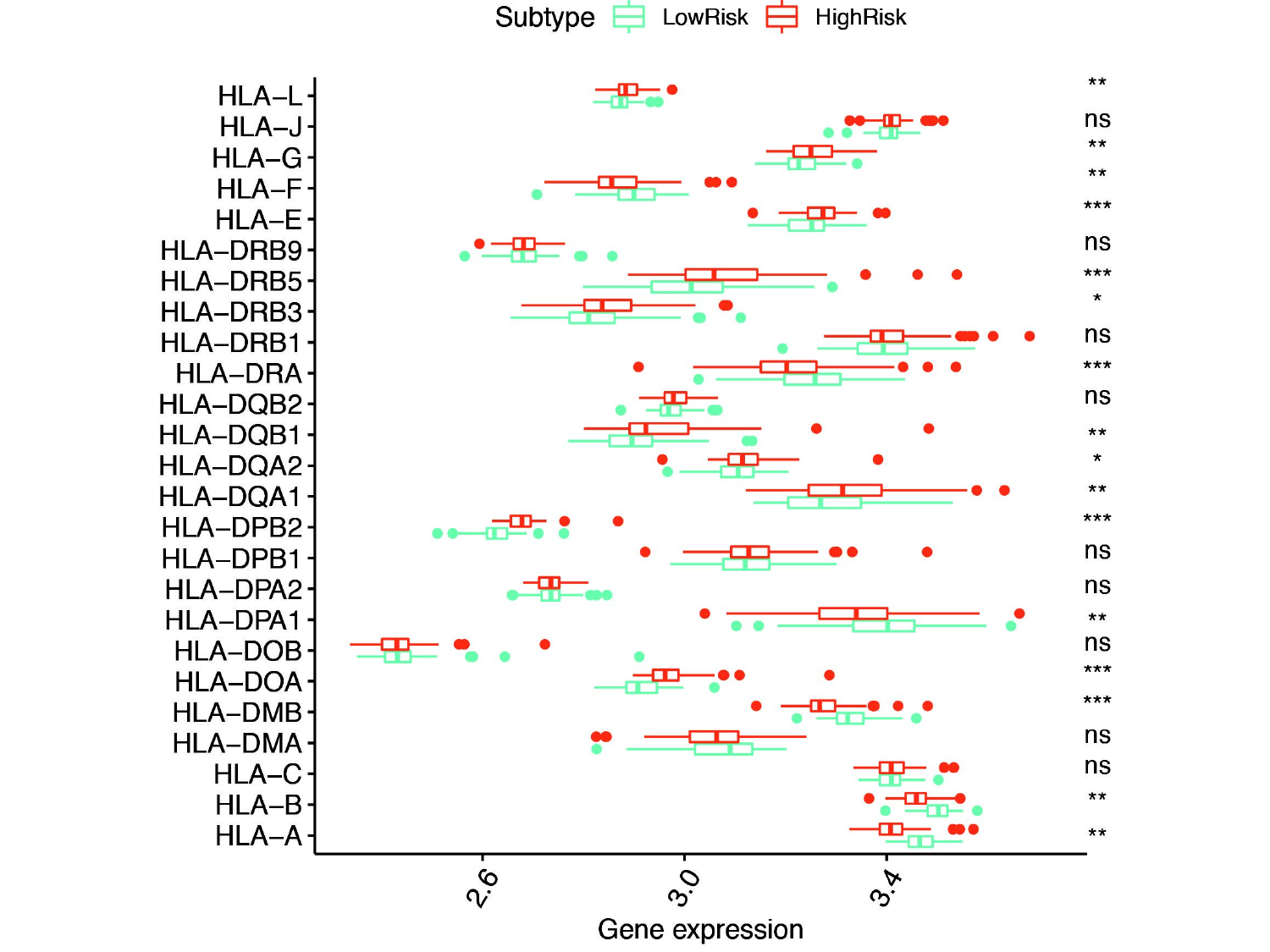

Supplement: Supplementary file 3 [file medi-102-e32741-s003.pptx]
